# A strengths-based approach to exploring diabetes management in an Indigenous minority population: A mixed methods study

**DOI:** 10.1371/journal.pone.0261030

**Published:** 2021-12-10

**Authors:** Kathleen Abu-Saad, Nihaya Daoud, Giora Kaplan, Arnona Ziv, Arnon D. Cohen, Daphna Pollack, Liraz Olmer, Ofra Kalter-Leibovici, on behalf of the Diabetes in the Arab Population in Israel (DAPI) Study Group

**Affiliations:** 1 Gertner Institute for Epidemiology and Health Policy Research, Sheba Medical Center, Ramat Gan, Israel; 2 Department of Public Health, Faculty of Health Sciences, Ben-Gurion University of the Negev, Beer Sheva, Israel; 3 Clalit Health Services, Tel Aviv, Israel; 4 Siaal Research Center for Family Medicine and Primary Care, Faculty of Health Sciences, Ben-Gurion University of the Negev, Beer Sheva, Israel; 5 Sackler Faculty of Medicine, Tel-Aviv University, Tel-Aviv, Israel; University of Copenhagen: Kobenhavns Universitet, DENMARK

## Abstract

Indigenous and other marginalized racial/ethnic minorities have poorer health status than majority populations, including higher rates of type 2 diabetes. These disparities have typically been addressed using a ‘deficit-based’ discourse that isolates disease management from the broader social, economic, political context and does not incorporate patient perspectives. We aimed to explore factors affecting glycemic control among Indigenous Arabs with diabetes in Israel using a strengths-based approach that centered participants’ knowledge of their context, needs, resources and strengths. We conducted an exploratory sequential mixed methods study, which included 10 focus groups (5 men’s, 5 women’s) and 296 quantitative in-person surveys. Participants with diagnosed diabetes were randomly drawn from the patient list of the largest healthcare service organization (survey response rate: 93%). Prominent and interconnected themes emerged from focus group discussions, including: diet, physical activity, and social, economic, mental/psychological and political stress. The discussions raised the need for adapting diabetes management approaches to incorporate participants’ communal, physical and psychological well-being, and socioeconomic/political realities. The connections between these factors and diabetes management were also reflected in multivariable analyses of the survey data. Women (OR: 2.03; 95% CI: 1.09–4.63), people with disabilities (OR: 2.43; 95% CI: 1.28–4.64), and unemployed people (OR: 2.64; 95% CI: 1.28–5.44) had higher odds of economic barriers to diabetes management. Furthermore, female sex (OR: 2.26; 95% CI: 1.25–4.09), unemployment (OR: 4.07; 95% CI: 1.64–10.10), and suboptimal glycemic control (OR: 1.20, 95% CI: 1.03–1.41 per 1-unit increase in HbA1c) were associated with moderate-to-severe depressive symptoms. A pro-active, team-based healthcare approach incorporating Indigenous/minority participants’ knowledge, experience, and strengths has the potential to improve individuals’ diabetes management. Healthcare services should be structured in ways that enable providers to listen to their patients, address their key concerns, and foster their strengths.

## Introduction

Indigenous and other marginalized racial/ethnic minorities have poorer health status than majority populations, including higher rates of type 2 diabetes, and diabetes-related complications and mortality [1–3]. In addressing these disparities, healthcare and government policy makers have typically used a deficit-based discourse that frames the problem as a deficiency, lack or failure of the Indigenous/minority population in comparison to a utopian non-Indigenous/majority ideal [4–6].

Such deficit-based approaches have been criticized for placing primary responsibility for the deficits with the affected individuals or communities (e.g., poor health literacy, poor health behaviors), while overlooking the contribution of inequitable socioeconomic structures to creating and perpetuating these deficits. When policy efforts to alleviate inequities are dominated by narratives of failure and dysfunction, the disadvantaged communities themselves are often positioned as the problem to be fixed. This renders their resilience and agency invisible, and negates their role as producers/co-producers of solutions [4].

Strengths-based approaches to addressing health and health disparities have emerged in Indigenous and other racial/ethnic minority communities as a counterbalance to deficit discourses [4, 7–9]. Strengths-based methods move the research focus from characterizing ‘problems’, to identifying the strengths of individuals and communities; and building upon their cultural assets, knowledge and resilience as avenues for action [4, 10]. They do not deny or disregard health disparities; but rather root them in the current and historical social, political, economic and cultural context; and identify the need for changes in systemic barriers and social determinants [4, 7, 10, 11]. Strengths-based methods aim to shift power to Indigenous and other marginalized communities by centering research on their concerns, and privileging their worldviews and ways of generating and sharing knowledge through traditional methods such as storytelling [4, 7, 12–15].

We adopted a strengths-based approach in the mixed methods study on Diabetes in the Arab population in Israel (DAPI), which explored factors affecting glycemic control among members of the Indigenous Arab minority with type 2 diabetes. The Indigenous Arab population of Palestine experienced disruption, displacement and dispossession of land and other resources when the state of Israel was established in 1948 [16–19]. It was transformed into a minority in the new Jewish state, and subsequently underwent separate and unequal developmental trajectories in education, employment and local infrastructure and resource allocation [16, 17, 20, 21]. This created numerous levels of structural disadvantage, including social, economic, political, and geographic marginalization [17, 19, 22]. Currently this Indigenous community comprises 20.9% of the total Israeli population [23]. It has a poverty rate of 50.3%, as compared to the national poverty rate of 21.2% (after governmental poverty alleviation transfers) [24], and a high level of residential segregation [25]; both of which negatively impact health [17, 25]. Arabs exhibit higher age- and sex-adjusted prevalence (18.4%) and annual incidence (2.9%) rates of adult-onset diabetes than the majority Jewish population (10.3% and 1.7%, respectively) [2]. They also exhibit younger age at diabetes presentation, suboptimal glycemic control, and higher diabetic complication and cause-specific mortality rates [3, 6, 26, 27]. These diabetes disparities exist despite the presence of national health insurance, community-based primary healthcare clinics, and regional secondary and tertiary healthcare facilities. Notably, however, most Arabs live in peripheral regions of the country that have poorer access to secondary- and tertiary-level services [28].

In the current article, we employed a strengths-based analytical approach to synthesize the DAPI study participants’ perspectives on the context, challenges and unmet needs relevant their day-to-day diabetes management and well-being; and their community’s strengths and resources for improving diabetes management.

## Methods

### Research design

We selected a sequential mixed methods design for this exploratory study because, in line with a strengths-based approach, we wanted to first elicit and center the participants’ perspectives, experiences and insights, and then further evaluate these findings in a larger, representative sample of Arab adults with diabetes. We used focus groups with open-ended questions in the first phase of the study to enable participants to take the lead in determining the most pressing concerns and issues. We then synthesized and utilized these data to shape the questionnaire for the cross-sectional quantitative data collected in the second phase of the study [29]. This design ensured that the quantitative instrument reflected the concerns and perspectives that arose in the focus groups, and enabled us to evaluate their generalizability in a representative patient sample. In the quantitative phase, we aimed to interview 300 participants with diabetes from five communities (60 participants per town/village), based on previous research [30] indicating that this would provide 85% power to detect differences in diabetes self-management behaviors by measures of participant strengths (e.g., 1 standard deviation increase in self-efficacy score) with an odds ratio of 1.46, with a two-tailed alpha of 0.05.

### Sampling frame and recruitment of study participants

The study sample was drawn from five (of 122) Arab communities that were purposively selected to reflect the range of geographic and socioeconomic variation of the Arab population in Israel (one city and village in the northern region, one city and mid-sized town in the central region, and one city in the southern region). The study was conducted in cooperation with Clalit Health Services (CHS), which provides healthcare coverage to approximately 70% of the Arab population [31]. Participants for both phases of the study were selected from a list provided by CHS of all patients in the study locality clinics with a diagnosis of diabetes (n = 7847). For the qualitative phase, a list of names was randomly drawn from the patient pool using a central computer-generated randomization process, stratified by town. The study coordinator contacted participants sequentially to invite them to participate in the focus groups. For the quantitative phase, another list of names was randomly drawn, excluding those in the patient pool for the focus groups. The quantified phase sample list was also stratified by town, and potential candidates were contacted sequentially. For candidates who refused to participate or could not be traced, an alternate was selected from the list, matched by town, sex and age group.

The study was approved by the institutional ethics committee (Helsinki Committee) of Sheba Medical Center. Participants in both study phases signed an informed consent form prior to participation.

Inclusion criteria were: 1) medical record diagnosis of diabetes, 2) aged 25–64 years, 3) community-dwelling (non-institutionalized) member of CHS in one of the 5 study towns, 4) and mentally/intellectually/physically capable of participating in a focus group/responding to survey questions. Exclusion criteria were: 1) erroneous medical record diabetes diagnosis, and 2) change in healthcare service organization, town of residence, or community-dwelling status.

### Data collection and analysis

#### Qualitative phase

The core research team (ND, OKL, GK, KA), Arab focus group moderators and Arab study coordinator met to develop the focus group discussion guide using established methods [32, 33]. The discussion guide began with a vignette about a typical man or woman with diabetes, and then posed a number of open-ended questions about what could facilitate or prevent the hypothetical person from reaching adequate glycemic control (see S1 Appendix for the focus group discussion guides). In line with a strengths-based approach, the use of broad, open-ended questions enabled the participants to direct the discussion and center it on the issues, needs, strengths and life experiences of key importance to them.

Ten focus group discussions were conducted in Arabic by trained Arab moderators (one among women and one among men in each of the five study localities) in local CHS clinics. The groups included from three to 15 participants. Prior to the focus group discussions, participants completed a short, anonymous demographic questionnaire. All focus groups were audio-recorded with the permission of the participants. The focus group recordings were transcribed in the original language (Arabic).

#### Quantitative phase

To prepare the survey for the quantitative phase, we reviewed a broad range of existing questionnaires related to diabetes care and experiences of patients with diabetes. We evaluated these instruments against the synthesis of the focus group data to select, adapt, and/or develop questions that best captured the key issues/themes that emerged from the focus groups. This enabled triangulation (e.g., cross-checking data from multiple sources) of the qualitative and quantitative data. The questionnaire was translated into Arabic by a certified translator, and the translation was checked by the Arab researcher in the core team (ND). The questionnaire solicited information on socio-demographic parameters, diabetes therapy, general and diabetes-specific health status, depression symptoms (using the Patient Health Questionnaire-9 (PHQ-9) [34]), self-management behaviors, healthcare service utilization, quality of care (using questions based on the Patient Assessment of Chronic Illness Care (PACIC) questionnaire [35]), and patient perspectives on adequacy of diabetes self-management education and barriers to/benefits of maintaining good glucose control. The questionnaire was piloted among 15 people with diabetes from an Arab town not included among the study towns. A number of questions were modified based on feedback from the pilot to improve the construct validity (e.g., ensure that questions measured the concepts they were intended to measure).

Of the survey respondent candidates contacted from the randomly drawn list of diabetic patients in the study town CHS clinics (n = 345), 49 candidates were not interviewed. Most (n = 26) were ineligible due to mental or physical health issues preventing participation, erroneous classification as diabetic, change to a different health care service provider, residence in a permanent care facility, death, or moved out of study town. The remainder were eligible candidates who could not be traced (n = 9); or refused to participate (n = 14). The final sample included 296 participants, representing a response rate of 92.8% among eligible candidates. Most participants (62.8%) were interviewed in their homes, while 37.2% were interviewed in their local clinics. The interviews were conducted from July 2012 to June 2013.

#### Analysis

For the qualitative data, we used a phenomenology methodologic approach [36] to analyze the focus group participants’ perceptions and lived experience of diabetes management. This aligns with a strengths-based approach, as it prioritizes identifying meaning and patterns from the data shared by participants, rather than imposing externally-determined theories and meanings on the data [4, 36]. A coding scheme was developed through an iterative process, in which initial independent readings of the Arabic transcripts/replay of the recordings by two team members (ND and KA) yielded a list of themes that emerged in the discussions. In addition, two transcripts (one women’s and one men’s) were translated into Hebrew and independently read by all core team members to identify themes. Emergent themes from all 10 focus groups were consolidated by ND and KA and then discussed with the rest of the team to identify the key issues for inclusion in the survey and further exploration.

During the course of the analysis, we noted the potency of numerous intact narratives to elucidate the intersectionality of the key issues raised in the focus groups. We therefore saw the value of preserving and presenting this data as intact narratives. Although this differs from the traditional method of presenting qualitative findings, it better aligns with strengths-based [4] and critical interactionist methodologies [37]. These methodologies center the experience and worldview of the participants, and prioritize the speakers/storytellers, rather than the researchers, in defining the meaning and purpose of the information they share, and the ways in which key issues are interconnected [4, 13–15, 37] (see S3 Appendix for more details on these methodologies).

In line with a strengths-based approach, the quantitative analyses centered on the issues of importance that arose from the focus groups, with the aims of triangulating the quantitative and qualitative data, evaluating the generalizability of the focus group findings, and exploring multivariable associations. Participant socio-demographic characteristics, health-status, and healthcare and self-management experiences were explored by sex in univariate analysis using chi-square or Fisher’s exact tests for categorical variables and the Wilcoxon test for continuous variables, with a threshold for significance of P<0.05. Factors associated with key issues raised in the focus groups (nutrition support/dietician consultation, physical activity, economic barriers, depression) were further evaluated in multivariable logistic regression models. All models controlled for sex and age, and other potentially relevant co-variates were entered using the forward-stepwise method, with a P-value <0.05 as the threshold for retention. All quantitative analyses were performed using SAS (version 9.4, SAS Institute Inc., Cary, NC).

## Results

Focus group participant characteristics are summarized in S1 Table in S1 File. They had a median of 8 years of education, a median age of 55 years at the time of the study (range: 33–64 years), and a median age of 45 years (range: 17–63 years) at time of diabetes diagnosis. Over 90% were taking oral hypoglycemic agents for diabetes treatment, while 44% of women and 63% of men were taking insulin.

The demographic profile of the survey respondents was similar to that of the focus group participants in age, marital status, and education (Table 1). Unemployment rates were high (89.8% and 59.6% among women and men, respectively), with 70.8% of unemployed men reporting being unemployed due to physical disability/illness. More than 50% of respondents were obese (BMI≥30.0 kg/m^2^). Median diabetes duration was 9.9 years. Half of the sample had Hemoglobin A1c levels ≥8.0%. Over half of the respondents (52.4%) were treated with insulin, with or without oral hypoglycemic agents (OHA), and 45.3% were treated with OHA alone. They took a median (IQR) of 6 (4–9) medications for chronic conditions. Over 80% had ≥2 chronic diseases in addition to diabetes, and ~50% reported that they were disabled. More women than men (45.5% vs. 24.8%, respectively) were identified with moderate-to-severe depressive symptoms (PHQ-9 score≥10) (Table 1).

**Table 1:** Selected characteristics of the DAPI survey respondents by gender (n = 296).

	Total (n = 296)		Women (n = 187)		Men (n = 109)		P^a^
**Demographics**							
Age *median (IQR)*	57	(51–63)	58	(51–64)	57	(50–63)	0.286
Marital status, *n (%)*							
Married	239	(80.7)	132	(70.6)	107	(98.2)	<0.001
Single/divorced/widowed	57	(19.3)	55	(29.4)	2	(1.8)	
Years of education, *median (IQR)*	8	(4–11)	8	(0–9)	9	(8–12)	<0.001
Currently unemployed, *n (%)*	233	(78.7)	168	(89.8)	65	(59.6)	<0.001
Highest occupational status level of participant or spouse score (range 1–9; higher score = lower occupational status), *median (IQR)*	9	(7–9)	9	(9–9)	8	(6–9)	<0.001
**Health status**							
BMI, kg/m^2^, *median (IQR)*	32.0	(28.1–36. 6)	32.5	(29.1–36. 9)	30.9	(27.1–35.0)	0.010
Age at DM diagnosis *median (IQR)*	45	(38–52)	46	(38–53)	44	(37–50)	0.154
DM duration, y *median (IQR)*	9.9	(6.3–15.3)	9.7	(5.9–15.6)	10.8	(6.6–15.0)	0.493
HbA1c, %, *median (IQR)*	8.0	(7.0–8.9)	7.9	(6.9–8.7)	8.2	(7.3–9.4)	0.038
DM therapy, n (%)							0.179
Diet alone	7	(2.4)	6	(3.2)	1	(0.9)	
Oral hypoglycemic agents alone	134	(45.3)	90	(48.1)	44	(40.4)	
Insulin with or without oral hypoglycemic agents	155	(52.4)	91	(48.7)	64	(58.7)	
2 or more chronic conditions (in addition to DM), n (%)	235	(79.4)	146	(78.1)	89	(81.7)	0.436
No. of meds median (IQR)	6	(4–9)	6	(4–9)	6	(4–8)	0.656
Disability, *n (%)*	149	(50.3)	99	(52.9)	50	(45.9)	0.241
Self-rated health status as poor, *n (%)*	200	(67.6)	131	(70.1)	69	(63.3)	0.231
Depression symptoms (PHQ-9 score ≥ 10), *n (%)*	112	(37.8)	85	(45.5)	27	(24.8)	<0.001
Cigarette smoking, *n (%)*							<0.001
Never	184	(66.7)	15	(83.3)	39	(38.2)	
Current smoker	52	(18.8)	18	(10.3)	34	(33.3)	
Past smoker	40	(14.5)	11	(6.3)	29	(28.4)	

DAPI Diabetes in the Arab population in Israel, IQR interquartile range BMI body mass index, DM diabetes mellitus, PHQ-9 Patient Health Questionnaire-9.

^a^P for chi-square or Fisher’s exact test for categorical variables, and for Wilcoxon test for continuous variables.

Most survey respondents received diabetes care from a family physician at primary healthcare clinics (84.8%), while 13.2% were treated by a diabetes specialist; and 91.6% had at least three visits to their diabetes care physician in the previous year. However, only 12.5% had visited a dietician in the previous year. The vast majority considered medications (95.6%), regular diabetes follow-up visits to a doctor/nurse (87.2%), and blood tests at the clinic (88.5%) to be important to their glycemic control. Most also reported receiving language-congruent (90.2%) and culturally-congruent (93.6%) care from their physicians. Language-congruence with nurses was lower (71.2%), but cultural congruence was high (89.5%). However, language- and cultural-congruence with dieticians, among those who had ever visited a dietician, was lower (52.5% and 65.9%, respectively) (S2 Table in S1 File).

The most prominent factors that focus group participants raised as affecting their day-to-day efforts to achieve glycemic control included: diet, physical activity, and social, economic, mental/psychological and political stress. We present intact participant narratives/discussions that illustrate the interconnectedness of these issues to their broader social, environmental and healthcare provision contexts. Isolated/additional quotes from the focus groups that illustrate varied aspects within the main themes are presented in S2 Appendix.

### Lifestyle

In response to the questions about what could best help a person with uncontrolled diabetes, focus group participants discussed lifestyle most extensively. Similarly, 91.6% and 83.5% of the survey respondents agreed that patients could improve their glycemic control through adherence to diet and physical activity recommendations, respectively (S3 Table in S1 File).

#### Diet: “Food is the main thing; if you eat everything, no doctor can help you.”

Focus group participants expressed broad consensus about the importance of lifestyle to both the development and the control of diabetes, and discussed the many factors that influenced it, including: traditions, lifestyle transitions, family, society, economics and politics (S2 Appendix. In one group, women reminisced about how active and healthy their traditional life was, and connected its disappearance to the emergence of chronic diseases:

W4-1: The older generation really worked hard; and there was no diabetes or high blood pressure. In the past, we used to harvest [wheat and barley] by hand, pile up the straw, and load it up on camels. And then we baked bread, and then washed our children’s clothes by hand, and there were no ‘pampers’, only cloth diapers. What have women today seen of this? Nothing!
W4-2: In the past, we didn’t have diseases like we have now.
W4-3: The food they had was all grown by them, everything; and they ate what they produced. It was different then….
Moderator: So life was better in the past?
W4-1: By God, it was better, and we’d never heard of these diseases….

The survey respondents indicated that the dietary management support they received from the healthcare system was limited. Although the national health insurance law in Israel entitles a person with diabetes to multiple meetings with a dietician per year at no cost [38], 54% of the survey respondents had never visited a dietician, and another 33% had not seen a dietician in the past year (S2 Table in S1 File). Only 16% reported getting their diet management knowledge from a dietician; compared to 40% from a healthcare provider without nutritional expertise (e.g., doctor, nurse), and 44% from family or community members, media sources, or were without a source of dietary information (S3 Table in S1 File). In a multivariable logistic regression model controlling for age, sex, education, diabetes duration, HbA1c value and insulin use, the factor that most increased the odds of ever visiting a dietician was being treated by a diabetes specialist rather than family physician (adjusted OR: 3.89, 95% CI: 2.31–6.58) (Table 2A and S4 Table in S1 File). This indicated low odds of dietician consultations for the vast majority of diabetic patients (85%) who were treated by family physicians.

**Table 2:** Multivariable logistic regression models of factors associated with a) ever having a dietician consultation; b) meeting the leisure physical activity recommendation; c) reporting economic barriers to any aspect of diabetes management; and, d) moderate-to-severe depression symptoms among DAPI survey respondents (n = 296).

Factor	Odds Ratio	95% Confidence Interval	P
A. Outcome: Ever having a dietician consultation			
Age (per 10-y increment)	0.93	0.69–1.24	0.604
Female vs male	1.25	0.73–2.16	0.417
Education, years (per 5-y increment)	1.42	1.05–1.92	0.023
DM duration (per 5-y increment)	1.22	1.01–1.47	0.041
Ever visited DM specialist: yes vs no	3.89	2.31–6.58	<0.001
C-statistic: 0.71			
B. Outcome: Meeting the leisure physical activity recommendation*			
Age (per 10-y increment)	0.91	0.60–1.39	0.666
Female vs male	1.97	0.84–4.59	0.118
Years of education (per 5-y increment)	1.84	1.08–3.16	0.026
DM duration (per 5-y increment)	0.61	0.42–0.90	0.012
No physical limitations to exercising	7.78	2.99–20.23	<0.001
Glycemic control not disrupted by za’al**	2.64	1.07–6.52	0.036
C-statistic: 0.84			
C. Outcome: Reporting economic barriers to any aspect of diabetes management			
Age (per 10-y increment)	0.97	0.94–1.01	0.119
Female vs male	2.03	1.09–3.80	0.026
Have disability: yes vs no	2.43	1.28–4.64	0.008
Currently unemployed vs employed	2.64	1.28–5.44	0.007
C-statistic: 0.71			
D. Outcome: Moderate-to-severe depression symptoms (PHQ-9 score≥10)			
Age (per 10-y increment)	0.74	0.53–1.02	0.067
Female vs male	2.26	1.25–4.09	0.007
Have disability: yes vs no	2.71	1.54–4.77	0.001
Currently unemployed vs employed	4.07	1.64–10.10	0.003
DM duration (per 5-y increment)	1.36	1.11–1.66	0.003
HbA1c test result (per 1% increment)	1.20	1.03–1.41	0.022
C-statistic: 0.75			

DAPI Diabetes in the Arab population in Israel, DM diabetes mellitus, SBGM self blood glucose monitoring, PA physical activity.

*≥2.5 hrs moderate-to-vigorous activity/wk.

**Sadness/anger/distress.

Of those who had visited a dietician, one-third did not think that the dietician’s advice adequately took their family and cultural context into account (S2 Table in S1 File). Others reported failing to achieve better glycemic control, or to adhere to the dietary advice of dieticians for reasons described in S2 Appendix.

Mainstream Western healthcare approaches to nutritional therapy for a person with diabetes focus on treating the individual. However, food is primarily consumed in the context of family and social gatherings, and the study participants related their dietary management efforts to this reality (S2 Appendix). Nearly 40% of survey respondents reported that the need to eat differently from the rest of the family was a barrier to adhering to diabetes dietary recommendations (S3 Table in S1 File). Most female participants (both focus group and survey respondents) were homemakers who held primary responsibility for the preparation of food for the household, and the need to eat differently from the rest of the family was particularly challenging for them:

W4: …we want to eat our traditional foods, like lentil soup, and the traditional vegetable dishes, but our children don’t want to eat this! We cook lentils and burgul (cracked wheat), see, and the kids don’t eat it. So we go along with them, eating fried food, grilled meat…kebab, and all kinds of frozen things…. What is our generation to do? Am I supposed to cook 20 different dishes? Forget it; I make the food they want, and I eat it too, and that’s the end of it!!

Men also had issues with the family food environment, including being served foods that were not recommended, or being pressured to overeat by their wives (S2 Appendix).

Beyond the nuclear family, participants discussed the important role of food sharing in maintaining community relationships. Consequently, refusal to eat at social events/celebrations (e.g., weddings, funerals, births) represented a withdrawal from community. About 20% of survey respondents reported this as a barrier to adhering to dietary recommendations (S3 Table in S1 File), and focus group participants also discussed the issue:

M4: The weddings and social events are a problem. Friends without diabetes *wound* the person with diabetes with their hospitality. They insist on serving him sweet soft drinks, which actually hurts him, and him alone.

Focus group participants also discussed the detrimental effect of political and economic policies on the diet of their families and communities:

M1: One of the reasons that we have diabetes…is our internal political situation, which is different from the situation of the Jews….Our political situation in this country is designed to have us eating only rice and bread. So, from the time my son was small, every day he would eat at least 3–5 pita breads [a.n. the equivalent of 9–15 carbohydrate exchange portions]. Why? Because I need to find the cheapest thing in order to feed my 4 or 5 or 6 children. And even the father who has only 1 child has to feed him a lot of bread…. We get the kids used to eating a lot of rice and other [unhealthy] things, and even over-eating just to make sure they are full….We have to increase the awareness of the children today, to start prevention efforts at an early age to keep them from developing diabetes through food.

#### Physical activity: “A person has to ‘treat his own laziness’ and make himself exercise.”

Although study participants generally agreed on the importance of physical activity to glycemic control, few survey respondents (12.2%) reported achieving recommended levels of leisure physical activity (≥2.5 hr/wk of moderate-intensity activity) (S3 Table in S1 File). Focus group participants described common barriers to achieving these recommendations, such as musculoskeletal problems and other co-morbidities (S2 Appendix), which were confirmed by 65.8% of the female and 46.8% of the male survey respondents (S3 Table in S1 File).

Focus group participants also discussed the lack of infrastructure for engaging in leisure physical activity in their communities, many of which were built prior to the existence of automobiles (S2 Appendix); and 29.9% of the female and 19.3% of the male survey respondents affirmed this as a barrier (S3 Table in S1 File). As one woman described:

W1: I live in the center of the town…I tried to walk from the door of my house to the entrance of the neighborhood, which is about 300–400 meters, and it was impossible. The street [a.n. which has no sidewalks] was completely jammed with cars. When I went out, I was almost hit by a car….I can’t see out of one eye.…

This woman advocated for a sports club to provide a safe place for women to exercise, and also create a support group to address/alleviate other psycho-social needs that affected their diabetes management:

W1-1: We need a sports club where we can come and talk about our diabetes, and learn other kinds of exercises, especially for the women who can’t walk. Our town is so crowded, there is no place to exercise… ‥ I am demanding this from our clinic on behalf of all the women here! We want a club where we can come and exercise our arms and legs, and get our blood sugar to go down….
Moderator: Would women come if there were a sports club for them?
W1-1: Yes we would come; every one of us would come and forget our problems for a while. Believe me, our high blood sugar also comes from all of the stress and pressure we’re living with….
W1-2: If only we could go out for an hour or two every day, and change the atmosphere, and forget about all the worries around us and in our homes…
W1-1: Now when we meet up and sit together, the whole conversation is about diabetes, and we start saying, “come on, let’s go out and exercise…hey, do you want to exercise…” We discuss everything: food, our moods, the whole conversation is about diabetes!

A multivariable logistic regression model controlling for age, sex and diabetes duration indicated that a similar array of factors were associated with survey respondents’ physical activity behaviors. Odds of meeting the recommended level of leisure physical activity was higher among those with no physical limitations to exercising (OR: 7.78, 95% CI: 2.99–20.23), those not reporting that sadness/distress impacted their glycemic control (OR: 2.64, 95% CI: 1.07–6.52), and those with more education (OR: 1.84, 95% CI: 1.08–3.16 per 5-year increment) (Table 2B and S5 Table in S1 File).

#### Economic situation: “If you don’t have money…it raises your blood sugar to 1000!”

Participants in all focus groups discussed the impact of economic stress on their health and diabetes control:

M4-1: People have diabetes because they don’t have money. If there’s no money, but the kids are going to school and want money, and the wife wants money, you just….There’s no money, but there’s plenty of diabetes! [All the participants laugh.]
Moderator: Do you get upset if your wives spend money?
M4-1: Of course; we need to buy medicine, and the things the kids want, so we get upset. If you don’t have money, it has a negative effect on your diabetes.
M4-2: By God, it raises your blood sugar to 1000!

Across the study, participants confirmed having difficulties paying for the direct and ancillary costs of diabetes care (e.g., medication co-payments, travel to appointments, recommended foods and physical activity). Over two-thirds (67.4%) of female and 47.7% of male survey respondents reported problems affording medicine or diabetes supplies; and 84.5% and 66.1%, respectively, reported an economic barrier to one or more of the direct/indirect costs of care (S3 Table in S1 File). In multivariable logistic regression analysis, the odds of reporting economic barriers to some aspect of diabetes management were higher for women (OR: 2.03; 95% CI: 1.09–4.63), people with disabilities (OR: 2.43; 95% CI: 1.28–4.64), and unemployed people (OR: 2.64; 95% CI: 1.28–5.44) (Table 2C and S6 Table in S1 File).

Focus group participants grounded the impact of economics in the broader context of their lives:

M1-1: When I want to buy medications, it costs for 300 ILS for mine, 250 ILS for my wife’s, and 200 ILS for my disabled son’s, so I need 750 ILS a month. Should I lie to you? I would be a liar if I told you that I bought them all each month.
M1-2: The healthcare provider charges for medications are problematic…
Moderator: Are you saying that their calculations aren’t right?
M1-2: I’m saying that if my blood sugar is high, and I can’t afford the medicine, they should give it to me for free. That will prevent me from being hospitalized, which is much more expensive for them than the medicines. They raised the prices of the medicine, and I couldn’t afford it…I would go once to bring [only] my wife’s medicines; another [month] I didn’t bring any at all.

Other focus group participants discussed the varied contextual aspects (e.g., competing costs for housing, essential utilities, extended family needs) that exacerbated economic barriers, and their coping strategies in the face of inadequate resources (e.g., reducing medication dosage/frequency, taking medications from a family member with diabetes) (S2 Appendix).

#### Psychological stress: “Za’al [sadness/distress] destroys your diabetes control, like a gun shot. You must not get upset!”

Most participants considered psychological distress, as expressed by the Arabic term za’al, to be an important factor in diabetes management. Za’al can be translated, depending upon the context, as sadness, frustration, stress or anger. In the focus groups, women often discussed za’al in relation to stressful relationships within the family or close community, as well as economic stresses (S2 Appendix):

W3-1: A stress-free home atmosphere can help you balance your blood sugar.
W3-2: If your children constantly frustrate you, there is no chance your blood sugar will go down.
W4: I have children of all ages. I’m afraid that any day I might die and leave them. None of them is married yet. These worries make my blood sugar very high.

Men primarily related za’al to economic pressures:

M1: Za’al comes from not having enough money to meet the many needs and requests of the family, and keeps the blood sugar high….I have four children…all of them are young. I live on 1000 ILS a month, thanks be to God. That’s not enough for my medicine; I try not to think about it. But the truth is that my worries and za’al make my blood sugar high.

Most survey respondents (83.1%) indicated that za’al prevented them from achieving adequate glycemic control (S3 Table in S1 File). Over one-third (37.8%) had scores ≥10 on the PHQ-9 questionnaire, indicating moderate-to-severe depressive symptoms; and this percentage was significantly higher among women (45.5%) than men (24.8%) (Table 1). In line with the focus groups, a multivariable logistic regression model indicated that female sex (OR: 2.26; 95% CI: 1.25–4.09), disability (OR: 2.71; 95% CI: 1.54–4.77), unemployment (OR: 4.07; 95% CI: 1.64–10.10), diabetes duration (OR: 1.36, 95% CI: 1.11–1.66 per 5-year increment) and suboptimal glycemic control (OR: 1.20, 95% CI: 1.03–1.41 per 1-unit increase in HbA1c) were concurrently and independently associated with moderate-to-severe depressive symptoms (Table 2D and S7 Table in S1 File).

#### Socio-political context

Many study participants reported that discrimination (61.8%) and political stress (39.5%) negatively affected their diabetes control (S3 Table in S1 File and S2 Appendix), adding another layer of complexity to their diabetes management efforts. Following a focus group discussion that demonstrated participants’ awareness of the many aspects of self-care important to good diabetes control, the moderator asked the participants why, if they knew this, did they not do it. One participant provided a multi-layered response that connected experiences of victim-blaming, infrastructure disparities, the socio-political milieu, and psycho-social pressures:

M1: I went to the hospital to visit a neighbor’s child with a burn injury. I found the [Jewish] department head grabbing the young Arab men and yelling at them, saying “You are Israeli Arabs; you were born in a civilized country…but you still aren’t advancing. Why not? I have 30 patients in my department with burns, breaks and other injuries…Of the 30, 7 are Jews and 23 are Arabs.” The father [of the hospitalized child] and his friends were silent; they could see that in every room there were 3–4 Arabs and maybe 1 Jew. So I came over to the department head and said, “I’m a visitor who came to see this burn patient. May I say something?” He agreed, and I said, “the reason is to be found among you [in the majority Jewish society]; if the young men here don’t know how to answer you, I have the answer. The first reason lies here,” and I asked him where he lived. He said in [Jewish city]. I said that when he goes home and his son asks to go outside to play with him or goes out by himself, he has a park with grass to run around and play in. But when I come home to [Arab city], and my son asks to play outside, the only place for him and the other children to play is in the road; so my son will break something, and your son won’t.
Moderator: Okay, but what’s the connection between this and diabetes?
M1: For me now, it is also political. I say, as an Arab citizen of Israel, the psychological pressures that I have to cope with are ten times greater than the pressures the Jewish population has.

## Discussion

This article provides an in-depth look at factors affecting diabetes management from the perspective of patients in an Indigenous minority population. Although they live in a high-income country with universal health insurance, diabetes control was sub-optimal among 50% of the study sample. The focus group discussions illuminated the necessity of managing diabetes within the broader context of patients’ lives, rather than in isolation from their communal, socioeconomic and political realities. These insights were confirmed by a representative sample of people with diabetes from the same communities.

This study took a strengths-based approach, using the experience and analysis of community members with diabetes as the starting point both for evaluating healthcare system successes and shortcomings, and for identifying community needs, strengths and solutions. It adds to the body of strengths-based approaches in the literature that serve to counterbalance and critique deficit-based approaches characterizing Indigenous and other marginalized minority patients as “problematic” or “non-compliant” [4, 6, 39].

Intact narratives from the focus group discussions illuminated the interconnectedness of socio-cultural, economic, psychological, healthcare system and political factors to diabetes management. We used the narratives to prioritize, elucidate and learn from the meanings, analyses and interconnections shared by the participants themselves. This differs from traditional qualitative analysis methods in which the researchers break the data down into small units, and then take the lead in regrouping, identifying themes and assigning meaning. Indigenous participant-researchers have objected to this type of qualitative data analysis because ‘breaking apart people’s stories’ forfeits the understanding of ‘the whole picture’ conveyed by the intact narrative [15]. Critical interactionist analytical approaches also use narratives to facilitate shifting the discourse away from the dominant academic/social voice and centering the perspectives of marginalized groups, so that ‘their lived experience becomes the vehicle through which new knowledge is not only created but also made relevant to the communities of concern’ [37:499]. Narrative- and storytelling-methods are increasingly being used in public health research [40–44] because they enable healthcare providers and policy makers to virtually experience, and thus better understand and address, the multi-level effects of social determinants on their patients’ lives, health, and healthcare experiences [13, 14, 42–45]. The results of the quantitative multivariable analyses also supported the connections described by focus group participants between socioeconomic, psychological, health, and healthcare system factors and the healthcare access, economic barrier, health behavior and mental health outcomes we examined. In the following sections, we highlight lessons from participant narratives that provide novel insights and suggest new directions for configuring diabetes healthcare and self-management in this Indigenous community.

### Healthcare system issues

The participant narratives and strengths-based methodology presented in this article support a healthcare delivery approach that aligns with patient-centered care. The methods we used also offer a practical model for concretely ‘centering the patient’. They invite providers to temporarily suspend the standard treatment protocols and their own judgements, and begin by listening to the patient. Patients have expertise and strengths to offer that include the description and analyses of their lived experiences, their resources, the barriers they face, and the support they need to overcome those barriers. Having listened to this, providers can better bring their expertise together with that of the patients’ to craft more personalized and effective diabetes management strategies.

Beyond the provider-patient relationship, DAPI participant perspectives raised several areas that would benefit from healthcare reform. In light of the intersecting economic, psychosocial, and structural barriers to diabetes management participants described, a pro-active, multi-disciplinary and cross-sectoral healthcare service approach is warranted. For example, although participants exhibited a strong awareness of the importance of diet to diabetes management, the odds of receiving a dietician consultation were high only for the minority of patients who were treated by a diabetes specialist. This is a reactive approach, as it typically occurs for those with sub-optimal glycemic control and more advanced disease [46]. Participants also expressed unmet needs in the area of social, psychological, and physical activity support. A proactive use of multidisciplinary teams that actively addresses and includes family and social contexts could potentially have a major impact on improving diabetes prevention and management.

In addition, the cost of copayments for medications is a well-documented access-to-care barrier that reduces the reach and effectiveness of Israel’s universal health insurance model [5, 6], and has not been adequately addressed, according to many of the study participants.

### Lifestyle issues

Numerous healthcare studies have positioned poor diet and sedentary lifestyles as inherent ‘deficits’ of Indigenous/marginalized communities that lead to higher diabetes rates [4, 6, 39]. In contrast, the focus group participants spoke of the transition they underwent—not of their own making—that separated them from a lifestyle in which nutritious food, physical activity and good health were inextricably intertwined. While the dominant population’s public health discourse has focused on the need to ‘introduce’ healthy lifestyle behaviors to the Arab population [5, 6, 26]; these participant’s memories firmly rooted healthy behaviors in their community’s Indigenous identity, making them instead traditions to be reclaimed in a new context. This is particularly critical in light of the entrance of Western junk food into the Arab diet, particularly among the youth [47]; which, as study participants discussed, ends up affecting the diet of the whole family [2, 48].

The political and economic contextualization that participants provided framed their families’ diets (and particularly what they could ‘count on filling up on’) not as a matter of individual choice; but rather as the consequence of a politically-constructed economic precariousness. Combined with the loss of land resources, this economic precariousness resulted in energy-dense, nutrient-poor food intake, and increased their community’s susceptibility to nutrition-related chronic diseases. The participants’ experience-based analysis makes the connection between government policies that were designed to maintain control over Arabs by keeping them economically weak [18–21, 48, 49] and their current health and nutritional status. Similar connections have been made in the international literature documenting associations between high intake of inexpensive, energy-dense foods (often government-subsidized or directly provided to Indigenous/low-SES populations) and high obesity and diabetes rates [50–53]. Thus, interventions aimed at getting people with diabetes to eat healthier diets must also address the economic and political realities that make it unfeasible for their families, or even for them as individuals, to comply.

The focus group narratives surrounding physical activity illustrated the many intersecting aspects of people’s lives that affect their lifestyle management efforts, including physical and infrastructure limitations, and needs for resources beyond the formal clinical context. A review of healthcare system interventions to reduce lifestyle-related risk factors in Indigenous Canadian communities found that their effectiveness was severely limited by structural barriers in the community (e.g., poor walkability) that could not be overcome simply by advising community members to adopt healthier behaviors [54]. Interventions co-designed by community members who intimately understand the context and barriers have the potential for greater success, as demonstrated by the Heart Health program developed with and for Australian Aboriginal people in the Derbil Yarrigan Aboriginal Health Service [55]. This program pragmatically addressed social determinants and provided a responsive, relationship-fostering, physically- and culturally-appropriate intervention through offering transportation, welcoming additional family members as the interest/need arose, and allowing flexible attendance that fit people’s lives. It has run for over 10 years and been consistently well-attended [55, 56]. Similarly, as some of the female focus group participants proposed, if local clinics provided neighborhood-based physical activity interventions, this would help overcome individual-level physical barriers and community-level structural barriers to exercising; and would simultaneously address women’s mental health and peer-support needs.

Healthy lifestyle interventions that focus on individual patients in isolation from their social and environmental context are challenging to implement in holistic, collective societies. The individual and the family are largely inseparable in collective societies, and suffer when the individual–family relationship is disrupted [57]. Healthcare system efforts to impose an individualistic outlook on patients in such contexts have had little success. An alternative approach in societies that prioritize social over individual obligations, could build on the strong social bonds to promote healthy lifestyle behaviors as social obligations on a community-wide basis. Community-designed and -led lifestyle interventions that engaged people of all ages (through clinics, schools, workplaces, community centers, food retailers, social media, etc.) would have the potential to promote diabetes management and prevention simultaneously, and to improve both their efficacy and cost-effectiveness. Support for such family-/socially-embedded approaches also emerged from a study among Arab Americans with diabetes [58].

### Economic and socio-political issues

In a community suffering from high poverty and unemployment rates, participants described how the impact of economic barriers on their lives and diabetes management encompassed and went beyond the issue of direct medical costs. Participants’ cost-benefit analyses, coping strategies in the face of inadequate resources, and contextualization of competing costs provide healthcare staff and policy makers with a more comprehensive and intersectional understanding of how economics impact their patients’ health.

The final narrative presented in the results exemplified both deficit-based and strengths-based approaches to understanding barriers to health and self-care. Viewing the over-representation of Arabs among hospitalized children out-of-context, a hospital department head identified the Arab population itself as ‘the problem.’ The focus group participant, however, used his lived experience to illuminate the underlying conditions contributing to the disparity, and to highlight critical, previously un-considered factors essential to eliminating it. This story was a direct response to the moderator’s question of why people with diabetes did not do what they knew they should. Although, on the surface, the story seemed unrelated to diabetes management, as a response to the moderator’s question, it implied that people with diabetes also found themselves being blamed for not implementing recommendations that ignored the broader context of their lives. Such an approach not only failed to contribute to eliminating disparities, it also politicized the discussion for the participant.

It is beyond the purview of healthcare providers to remedy the economic and political stressors or inadequate local infrastructure that negatively affect diabetes management. Nevertheless, such narratives help providers understand why putting responsibility on individual patients to overcome barriers not of their making and beyond their control indeed becomes victim-blaming [4, 12]. Understanding the historical and social contexts that have given rise to disparities in social determinants enables healthcare providers to engage with patients and community members to address or circumvent the disparities [4, 56, 59]. Metzl et al. recommended promoting health equity by educating clinicians about social inequality and training them to “consider and treat the upstream structural, social and environmental conditions that often underlie disease” [60:232]. The focus group narratives presented here illustrate the importance of such education; and demonstrate the expertise that patients from Indigenous/marginalized minority groups have to contribute to the education of clinicians as a result of their lived experiences and analyses of these conditions.

Similarly, grounded knowledge of patients’ economic and socio-political vulnerability can help policymakers move the concept of ‘health in all policies’ from an abstract mantra to concrete objectives for which to advocate in cross-departmental/ministerial committees addressing the social determinants of health [59]. For example, DAPI participants’ narratives and concerns can direct healthcare policymakers to prioritize investigating and eliminating the large disparity in the poverty rates of Arabs and Jews, which persists despite governmental mechanisms to alleviate poverty. Such policy initiatives would have tangible potential to improve the conditions within which people carry out their day-to-day diabetes management.

### Study strengths and limitations

The strengths of this study include use of a sequential, exploratory mixed methods approach, and triangulation of the qualitative and quantitative data. The quantitative study was based on a randomly selected sample of patients with diabetes from the main healthcare organization providing services to the Arab population, and had a high response rate. We applied a strengths-based analytical approach, which centered participants’ perspectives, and has not previously appeared in the local literature. Limitations of the study include its exploratory and cross-sectional design; which, though useful for generating hypotheses, prohibits drawing conclusions about sequential or causal effects. In addition, all of the focus groups and a minority of the quantitative interviews were conducted in participants’ local clinics. Although the study activities occurred in closed rooms including only study staff and participants, the setting may have inhibited full expression of ideas critical of healthcare providers/system.

## Conclusion

In conclusion, this strength-based study highlights the need to learn from people with diabetes in marginalized Indigenous/minority populations in order to understand their lived experiences and socio-cultural strengths. This approach opens new avenues for improving patients’ diabetes management and overall wellbeing, and increasing the appropriateness and effectiveness of healthcare service provision. Patients have essential expertise on what is and is not working in their diabetes care, on the types of support they need to address the key structural barriers that confront them, and on the individual and community strengths they have to offer. Healthcare services should be structured in ways that enable providers to listen to their patients, address their key concerns, and foster their strengths.

## Supporting information

S1 Appendix Supplemental Appendix 1. Vignettes for focus groups.
(PDF)

S2 Appendix Supplemental Appendix 2. Themes and representative quotes from focus groups.
(PDF)

S3 Appendix Supplemental Appendix 3. Story/narrative-based and critical interactionist methodologies for reporting and analyzing qualitative data.
(PDF)

S1 File Supplemental tables. (PDF)
