# Supplementary material for: A strengths-based approach to exploring diabetes management in an Indigenous minority population: A mixed methods study
Source: PLoS One. 2021 Dec 10;16(12):e0261030. doi: 10.1371/journal.pone.0261030 (PMC8664199; doi:10.1371/journal.pone.0261030)
Supplement: S1 Appendix — Vignettes for focus groups. (PDF) [file pone.0261030.s001.pdf]

Supplemental Appendix 1. Vignettes for focus groups

Men's focus groups - Fakhri

A. Fakhri is a 57 year-old man, who is married to Ilham and works in a cement block factory. He has 6 children between the ages of 15 to 34, and 9 grandchildren. Two years ago he visited his doctor with complaints of weakness. His blood tests showed that he had high blood sugar. His doctor told him that he had diabetes.

   Questions:
   1. What does Fakhri need to do to achieve better blood sugar levels?
   2. What or who can help him achieve better blood sugar levels?
   3. What prevents him from reaching better blood sugar levels?

B. Two years after Fakhri's diabetes was diagnosed, his blood sugar levels were still high. His doctor told him that his eyes had been damaged due to his diabetes, and also told him about other complications that could develop, even including the amputation of a foot/leg, God forbid. Fakhri is afraid that this could happen to him.

   Questions:
   1. Why do you think that Fakhri didn't succeed to achieve good blood sugar levels?
   2. What or who could help him achieve better blood sugar levels?
   3. What does Fakhri need to do to achieve better blood sugar levels?
   4. What prevents him from reaching better blood sugar levels?

Closing questions:

1. As we explained to you at the beginning of this meeting, the aim of this research is to help develop programs specifically developed for the Arab population to help people with diabetes to achieve better blood sugar balance. Do you have any suggestions to give us?
2. Is there anything else that anyone would like to add?

Women's focus groups - Siham

A. Siham is a 54 year-old woman, who lives in an Arab village, and whose husband, Salim, works in a cement block factory. She is the mother of 6 children between the ages of 15 to 34, and has 9 grandchildren. She went to school for 8 years, and has been a housewife throughout her adult life. Siham lives near to her husband's family. Two years ago she visited her doctor with complaints of weakness. Her blood tests showed that she had high blood sugar. Her doctor told her that she had diabetes.

   Questions:

   1. What does Siham need to do to achieve better blood sugar levels?

   2. What or who can help her achieve better blood sugar levels?

   3. What prevents her from reaching better blood sugar levels?

B. Two years after Siham's diabetes was diagnosed, her blood sugar levels were still high. Her doctor told her that her eyes had been damaged due to her diabetes, and also told him about other complications that could develop, even including the amputation of a foot/leg, God forbid. Siham is very much afraid that this could happen to her.

   Questions:

   1. Why do you think that Siham didn't succeed to achieve good blood sugar levels?

   2. What or who could help her achieve better blood sugar levels?

   3. What does Siham need to do to achieve better blood sugar levels?

   4. What prevents her from reaching better blood sugar levels?

Closing questions:

1. As we explained to you at the beginning of this meeting, the aim of this research is to help develop programs specifically developed for the Arab population to help people with diabetes to achieve better blood sugar balance. Do you have any suggestions to give us?
2. Is there anything else that anyone would like to add?
