# Supplementary material for: A strengths-based approach to exploring diabetes management in an Indigenous minority population: A mixed methods study
Source: PLoS One. 2021 Dec 10;16(12):e0261030. doi: 10.1371/journal.pone.0261030 (PMC8664199; doi:10.1371/journal.pone.0261030)
Supplement: S2 Appendix — Themes and representative quotes from focus groups. (PDF) [file pone.0261030.s002.pdf]

Supplemental Appendix 2. Themes and representative quotes from focus groups

Note: M1-M5 and W1-W5 represent the numbers of the men's and women's focus groups, respectively. Quotes including comments from multiple participants in the same focus group discussion are indicated, for example, by M1-1, M1-2, M1-3.

**Themes**	**Representative Focus Group Quotes**
**Diet**	***General***
	M4: Food is the main thing; if you eat everything, no doctor can help you.
	M1: Diabetes is like an infection that can either be prevented by a healthy diet from childhood, or caused by an unhealthy diet from childhood.
	W1: The increase in DM in the Arab community is from changes in the food - to fast foods, sweets, cola - and especially among the youth.
	W4-1: Today all of the food is garbage. In the past, we used to eat tomatoes grown on rainwater, we used flour [from the grain] we planted and harvested. Now all of it is grown on waste water, waste water! Of course we'll develop diseases from eating this food!<br>W4-2: Vegetables are grown on chemicals…<br>W4-1: They shoot chickens up with drugs to get them to grow so fast; they blow them up like balloons and they slaughter them within a week…. Cook a Jewish chicken raised on injections, and cook an Arab chicken raised naturally…and see how delicious the locally-grown chicken is!!
	Healthcare system dietary counseling
	W1: I stopped following the diet given by the dietician because I was always hungry, so I made up my own diet.
	W2: I dieted and exercised like the dietician told me to for 5 months, and I didn't lose 1 kilo, so I stopped and went back to the way I was eating before.
	M1: I have to decide on my diet by myself; the doctor can't provide me with useful advice for that.
	Individual context of diet management
	W3: I was told what to eat, but I don't do it. It's hard not to eat sweet things when they are in front of you every day.

Themes	Representative Focus Group Quotes
	M3: I love sweets and fruit, and I can't prevent myself from eating them while everyone around me is eating them.
	M3: When you have to say no to everything, it's just too hard.
	M5-1: I don't have self-control. I want to, but I eat sweets and drink tea with 3 teaspoons of sugar. Every day I buy *kanafeh* [a sweet, cheese-based dessert]. When I go to weddings, I eat and drink sweets while sitting next to my family physician.<br>M5-2: I do the opposite, and my HbA1c is 6.2%.
	Social/community context of diet management
	W3: You are standing in the kitchen all day; the kids are very demanding and it's hard to satisfy them all.
	W5: When we make food we have to try every dish.
	M2: Your wife shouldn't put things in front of you that you are not allowed to eat.
	M2: Arabs have this issue with waste. You don't want to waste food so you finish everything. Your wife wants to use up some food that was left over from yesterday, so she pushes it on you….
	M2: The food at weddings!!! And the host [father of the groom] will force you to eat by swearing that he will divorce his wife if you don't eat!
	M4: The weddings and social events are a problem. Friends without DM *wound* the person with DM with their hospitality. They insist on serving him sweet soft drinks, which actually hurts him, and him alone.
	Economic context of diet management
	M3: Diabetes is an expensive disease; every diet food is very expensive.
	W4: I eat what we have. I don't always have the things the dietician told me to eat like low-fat yogurt, tuna, and chicken breast. And the special diet bread is very expensive.
	W4: I made bread with *inkhala* [wheat bran] and locally-milled whole wheat flour instead of buying expensive whole wheat diet bread [from the Jewish supermarket]...and my blood sugar levels went down. But then I got tired of

Themes	Representative Focus Group Quotes
	doing this and went back to eating regularly, without any limitations, and my blood sugar went back up.
**Physical activity**	***Physical barriers***
	W3: You should walk if you don't have any other diseases or disabilities.
	W1: I can't exercise, so I trust in God.
	Infrastructure barriers
	W1: I live in the center of the town…I tried to walk from the door of my house to the entrance of the neighborhood, which is about 300-400 meters, and it was impossible. The street [a.n. which has no sidewalks] was completely jammed with cars. When I went out, I was almost hit by a car….I can't see out of one eye….That's why I'm telling you that if a place was opened up for us to exercise in, we would go to it.
	W3: We drive to [a nearby Jewish town] to walk.
**Economic stress**	***General***
	M3: What keeps Arab men from maintaining good diabetes control? The burden of the family that they carry, and of being the sole breadwinner.
	M3: If a person has enough to live on and to support the family, then he can succeed to balance his blood sugar.
	Moderator: And what about the celebrations, like weddings, do they affect your diabetes?<br>M3: Weddings* are good, but they require a lot of resources [money]. Among the Arabs, they require so much money that they can bankrupt you.<br>*Moderator: And that has an effect on your diabetes?*<br>M3: Of course it has an effect. I was invited to 7 weddings in a month. Where can a person get the money for that?<br>*[*Note: Weddings are very important, elaborate, and expensive events that typically go on for a week. Male heads of households and their adult sons are expected to attend weddings in the community and to give a large monetary gift that is then returned to them when they host a wedding/marry.]*

Themes	Representative Focus Group Quotes
	W5: Our husbands aren't working anymore; they're living on social security so there is no money anymore for getting out of the house or the village and getting a break.
	Medications
	M4: You do what you can, buy a little bit of food to eat, and a little bit of medicine, divided up, so you take a little of the diabetes medicine, and a few of the cholesterol pills, and a few of something else, and that's how you keep yourself going with what you've got.
	W2: If I can't afford to buy my pills, I go to my sister [who also has diabetes] and get a few pills from her.
	Moderator: Does it happen often that you aren't able to buy medicine?<br><br>W1-1: Yes, it does.<br><br>*Moderator: None of you is working, where do you get the money from?*<br><br>W1-2: My husband gets 2400 ILS from unemployment [allowance] every month, he can't work; he had [coronary] bypass surgery. How can that be enough for the house, water, electricity, and 300 ILS for his heart medicines? He has to have the medicines, and there isn't enough money for all of medicines for both of us.<br><br>W1-3: If I want to get all my medicines, I need 600 ILS… Once [I went without it and] I was hospitalized for 2 weeks. I told one of my friends that I don't want to tell my brother or my sister. I'd rather be hospitalized and not ask them to give me money for medicines! The situation is hard for everyone today. Nobody can give; everyone is relying on God's mercy these days...<br><br>W1-2: Some have families; some have children, some of whom want to get married, want to study, want to build, and on and on.<br><br>W1-1: But God is merciful, don’t complain….there are some who have money, and help their brothers and sisters.<br><br>W1-2: In every 1,000, you won’t find one like this.<br><br>*Moderator: Did you try to go to [your healthcare provider] and get medicines when you didn't have money?*<br><br>W1-2: They don't give us anything [without paying], not a shekel's worth.

Themes	Representative Focus Group Quotes
**Mental/psychological Stress**	***General***
	W1: You must not get upset. *Za'al* [distress/depression] destroys your diabetes control, like a gun shot. You must not get upset!
	Social triggers
	W5: She [the hypothetical patient in the vignette with high blood sugar] had trouble at home, maybe with her husband or her mother-in-law.
	Economic triggers
	M5: The family should play a positive role in a father's diabetes by not upsetting him and asking him for things he cannot provide.
**Political context**	***Service disparities in minority communities***
	W5-1: There is no dietician or diabetes specialist in our village; we have to go to [the closest urban Jewish town] for this, and most people don't have transportation.
	W5-2: They built a community center in the village, but it's empty, there are no programs there. They have to have activities for women, on cooking, on handcrafts, on exercise, on everything. But there are no programs and they say there is no budget for making programs.
