# Supplementary material for: A strengths-based approach to exploring diabetes management in an Indigenous minority population: A mixed methods study
Source: PLoS One. 2021 Dec 10;16(12):e0261030. doi: 10.1371/journal.pone.0261030 (PMC8664199; doi:10.1371/journal.pone.0261030)
Supplement: S3 Appendix — Story/narrative-based and critical interactionist methodologies for reporting and analyzing qualitative data. (PDF) [file pone.0261030.s003.pdf]

Supplemental Appendix 3. Story/narrative-based and critical interactionist methodologies for reporting and analyzing qualitative data

The primary analysis of the qualitative data in this article is based upon the use of extended intact narratives. This analytical approach draws upon methodologies developed by indigenous scholars, who use intact stories/narratives, in continuity with traditional practices, to transmit knowledge and holistic understandings of the issues studied [1-7]. Stories convey relational knowledge, and peoples' sharing of their experiences is an important method of teaching [3,6-8].

Critical interactionist methodology is another research approach that has emerged from work among marginalized communities. It similarly centers intact participant narratives in order to examine participants' experiences within the context of a broad range of social factors, structural factors, and power relations that influence their health and healthcare encounters [9,10]. This approach facilitates a deep exploration of the dynamics that perpetuate health disparities, and possible avenues for transforming those dynamics to achieve equity. By utilizing extended, intact participant narratives, it centers the perspectives of marginalized groups; rather than, as is typically done, the dominant academic voice [9].

Both of these methodological streams are critical of traditional qualitative data analysis methods, which break the data down into small units that are then assigned meaning by the researchers and regrouped into discrete (disconnected) themes. Indigenous researchers have explicitly objected to this process of 'breaking apart people's stories' because it dismantles a story's holistic coherence and its valuable information about how the issues, actors and environment are connected to each other [2,3]. Indigenous storytelling and critical interactionist methodologies instead use intact narratives as units of analysis in order to maintain the storyteller's interpretation of the story and the relationships within it [2,3,9].

References

1. Drawson AS, Toombs E, Mushquash CJ. Indigenous research methods: a systematic review. Int Indigenous Policy J. 2017;8(2). Available from: https://ir.lib.uwo.ca/iipj/vol8/iss2/5
2. Hallett J, Held S, McCormick AKHG, et al. What Touched Your Heart? Collaborative Story Analysis Emerging From an Apsáalooke Cultural Context. Qual Health Res. 2017;27(9):1267-1277.
3. Simonds VW, Christopher S. Adapting Western research methods to indigenous ways of knowing. Am J Public Health. 2013;103(12):2185-92. doi: 10.2105/AJPH.2012.301157.
4. Dickson M. "My work? Well, I live it and breathe it": The seamless connect between the professional and personal/community self in the Aboriginal and Torres Strait Islander health sector. BMC Health Serv Res. 2020;20(1):972. https://doi: 10.1186/s12913-020-05804-3
5. Wain T, Sim M, Bessarab D, Mak D, Hayward C, Rudd C. Engaging Australian Aboriginal narratives to challenge attitudes and create empathy in health care: a methodological perspective. BMC Med Educ. 2016;16:156.
6. Caxaj CS. Indigenous storytelling and participatory action research: Allies toward decolonization? Reflections from the Peoples' International Health Tribunal. Glob Qual Nurs Res. 2015;2:2333393615580764.
7. Rieger KL, Gazan S, Bennett M, Buss M, Chudyk AM, Cook L, Copenace S, Garson C, Hack TF, Hornan B, Horrill T, Horton M, Howard S, Linton J, Martin D,

McPherson K, Rattray JM, Phillips-Beck W, Sinclair R, Schultz ASH. Elevating the uses of storytelling approaches within Indigenous health research: a critical and participatory scoping review protocol involving Indigenous people and settlers. Syst Rev. 2020;9:257. doi: 10.1186/s13643-020-01503-6.

8. Jones B, Heslop D, Harrison R. Seldom heard voices: a meta-narrative systematic review of Aboriginal and Torres Strait Islander peoples healthcare experiences. Int J Equity Health. 2020;19(1):222. http://doi: 10.1186/s12939-020-01334-w
9. Dubbin L, McLemore M, Shim JK. Illness narratives of African Americans living with coronary heart disease: A critical interactionist analysis. Qual Health Res, 2017;27(4): 497-508.
10. Dubbin L, Burke N, Fleming M, Thompson-Lastad A, Napoles TM, Yen I, Shim JK. Social literacy: nurses' contribution toward the co-production of self-management. Glob Qual Nurs Res. 2021;8:2333393621993451.
