# Supplementary material for: A strengths-based approach to exploring diabetes management in an Indigenous minority population: A mixed methods study
Source: PLoS One. 2021 Dec 10;16(12):e0261030. doi: 10.1371/journal.pone.0261030 (PMC8664199; doi:10.1371/journal.pone.0261030)
Supplement: S1 File — (PDF) [file pone.0261030.s004.pdf]

Supplemental Tables

Supplemental Table 1. Selected characteristics of the DAPI focus group participants by gender

	Women (n=36)		Men (n=27)	
Age (y), *median (range)*	54	(33-64)	57	(40-64)
Married, N (%)	25	(69.4)	24	(88.9)
Years of education, *median (range)*	8	(0-14)	8	(0-18)
Currently unemployed, *N (%)*	35	(97.2)	21	(77.8)
Age at diabetes diagnosis (y), *median (range)*	45	(17-63)	44	(21-61)
Diabetes treatment, *N (%):*				
Oral hypoglycemic agents alone, *N (%)*	34	(94.4)	26	(96.3)
Insulin with or without oral hypoglycemic agents, *N (%)*	16	(44.4)	17	(63.0)

DAPI Diabetes in the Arab population in Israel

Supplemental Table 2. Health care provision among the DAPI survey respondents by gender (n=296)

	Total (n=296)		Women (n=187)		Men (n=109)		P[a]
**Healthcare provision**							
Primary physician providing DM care, *n (%)*							
Family physician	251	(84.8)	156	(83.4)	95	(87.2)	0.101
Diabetes specialist	39	(13.2)	29	(15.5)	10	(9.2)	
Other	6	(2.0)	2	(1.1)	4	(3.7)	
Three or more visits to doctor providing DM care in past year, *n (%)*	271	(91.6)	173	(92.5)	98	(89.9)	0.437
Dietician visits, n (%)							0.960
Never	161	(54.4)	101	(54.0)	60	(55.0)	
None in past year	98	(33.1)	63	(33.7)	35	(32.1)	
≥1 in past year	37	(12.5)	23	(12.3)	14	(12.8)	
Adequate SBGM performance,[b] *n (%)*	151	(59.9)	112	(59.9)	66	(60.6)	0.911
Have supplemental health insurance*, n (%)*	220	(74.3)	140	(74.9)	80	(73.4)	0.780
Meet leisure physical activity recommendation (≥2.5 hr/wk)	36	(12.2)	23	(12.3)	13	(11.9)	0.925
**Patients' perception of importance of healthcare provision**							
High perceived benefit to glycemic control of: n (%)							
Taking medications	283	(95.6)	179	(95.7)	104	(95.4)	0.352

	Total (n=296)		Women (n=187)		Men (n=109)		P[a]
Regular follow-up visits to doctor/nurse	256	(87.2)	158	(84.5)	100	(91.7)	0.231
Regular follow-up visits to dietician	190	(64.2)	116	(62.0)	74	(67.9)	0.445
Regular blood tests at clinic	262	(88.5)	162	(86.6)	100	(91.7)	0.200
**Accessibility of health care team, n (%)**							
Doctor speaks my language	267	(90.2)	165	(88.2)	102	(93.6)	0.136
Nurse speaks my language	212	(71.2)	141	(75.4)	71	(65.1)	0.059
Dietician speaks my language	155	(52.5)	99	(53.2)	56	(51.4)	0.759
Doctor gives treatment I can implement in my daily life	283	(95.9)	177	(95.2)	106	(97.3)	0.545
Doctor gives recommendations that take my family and cultural context into account	276	(93.6)	177	(90.9)	107	(98.2)	0.013
Nurse gives treatment I can implement in my daily life	269	(91.2)	165	(89.2)	104	(95.4)	0.065
Nurse gives recommendations that take my family and cultural context into account	264	(89.5)	160	(86.5)	104	(95.4)	0.015
Dietician gives recommendations that take my family and cultural context into account (n=134)	89	(65.9)	57	(66.3)	32	(66.7)	0.964

DAPI Diabetes in the Arab population in Israel, DM diabetes mellitus, SBGM self blood glucose monitoring

[a]P for chi-square or Fisher's exact test for categorical variables, and for Wilcoxon test for continuous variables

[b]Adequate SBGM defined as ≥1 time/d for those treated with insulin, and daily, weekly or intermittently for those treated with oral hypoglycemic agents.

Supplemental Table 3. Diabetes-related knowledge, skills and resources among DAPI survey respondents by gender

	Total (n=296)		Women (n=187)		Men (n=109)		P
**Patient actions that can improve glycemic control, n (%)**							
Regular physical activity	247	(83.5)	158	(84.5)	89	(81.7)	0.112
Adherence to recommended diet	271	(91.6)	169	(90.4)	102	(93.6)	0.221
SBGM	252	(85.2)	152	(81.3)	100	(91.7)	0.051
**Lifestyle resources and barriers, n (%)**							
Diet							
Main source of dietary knowledge							0.118
Dietician	47	(15.9)	30	(15.6)	17	(16.0)	
Other member of health care team (doctor, nurse)	116	(39.2)	63	(33.7)	53	(48.6)	
Family/community members	51	(17.2)	36	(19.3)	15	(13.8)	
Various media sources	41	(13.9)	30	(16.0)	11	(10.1)	
None	41	(13.9)	28	(15.0)	13	(11.9)	
Difficult to find special dietetic foods locally	102	(34.7)	57	(30.8)	45	(41.3)	0.068
Recommended diet for DM not filling	152	(54.3)	100	(54.3)	52	(49.1)	0.385
Recommended diet for DM not tasty	160	(55.0)	108	(58.7)	52	(48.6)	0.095
Need to eat differently from the rest of the family is a barrier to adhering to a DM diet	116	(39.6)	78	(42.2)	38	(35.2)	0.239
Difficult to keep DM diet when always preparing food for the rest of the family (women only)	--	--	77	(41.6)	--	--	

	Total (n=296)		Women (n=187)		Men (n=109)		P
Uncomfortable refusing hospitality when offered food and drink that isn't part of the DM diet	57	(19.3)	35	(18.7)	22	(20.2)	0.758
Physical activity							
Health problems barrier to doing PA	174	(58.8)	123	(65.8)	51	(46.8)	0.001
Dislike doing sports/physical activity	81	(27.5)	52	(28.0)	29	(26.6)	0.802
Other obligations (e.g., family, work) prevent doing sports/physical activity	76	(25.7)	54	(28.9)	22	(20.2)	0.099
Conditions of built environment are a barrier to doing PA	77	(26.0)	56	(29.9)	21	(19.3)	0.043
**Economic resources and barriers, n (%)**							
Lack resources for doing sports/leisure physical activity	130	(43.9)	94	(50.3)	36	(33.0)	0.004
Lack resources for buying recommended foods for DM management	140	(47.5)	100	(53.5)	40	(37.0)	0.007
Prevented sometimes/often in past year by economic situation from:							
Buying medications	102	(34.5)	67	(35.8)	35	(32.1)	0.516
Buying supplies for DM care (e.g., SBGM test strips, lances)	103	(34.8)	66	(35.3)	37	(33.9)	0.814
Making visits to the dietician	106	(35.8)	70	(37.4)	36	(33.0)	0.446
Making co-payments for specialist visits/services	69	(36.8)	49	(26.2)	20	(18.3)	0.123
Paying travel expenses for various clinic visits	109	(36.8)	73	(39.0)	36	(33.0)	0.301
Paying to visit private physicians	148	(50.0)	102	(54.5)	46	(42.2)	0.041
Obtaining dental care	150	(50.7)	106	(56.7)	44	(40.4)	0.007

	Total (n=296)		Women (n=187)		Men (n=109)		P
Lack of money for buying medicines/supplies for DM self-management worsens my glycemic control	143	(48.3)	104	(55.6)	39	(35.8)	0.001
Economic distress/ request help for:							
Purchasing medicines/supplies for DM self-management	178	(60.1)	126	(67.4)	52	(47.7)	0.001
Any aspect of DM care (e.g., medications, health/dental care and associated travel costs, diet, PA)	230	(77.7)	158	(84.5)	72	(66.1)	<0.001
**Psychosocial-level, n (%)**							
Emotional state/motivational issues							
Sadness/stress /anger prevent maintaining adequate glycemic control	245	(83.1)	163	(87.2)	82	(75.9)	0.013
Avoidance of stress/distress/anxiety can improve glycemic control	269	(90.9)	174	(93.1)	95	(87.2)	0.116
"Getting out of the house and forgetting all your problems" can improve glycemic control	224	(75.7)	142	(75.9)	82	(75.2)	0.852
Social obligations and support							
Family tensions/pressures prevent maintaining adequate glycemic control	232	(78.6)	157	(84.4)	75	(68.8)	0.002
Family obligations (e.g., weddings, family gatherings) prevent maintaining adequate glycemic control	138	(46.6)	97	(51.9)	41	(37.6)	0.018
Have no one to talk to about the difficulties of having DM	70	(23.7)	49	(26.2)	21	(19.3)	0.176

	Total (n=296)		Women (n=187)		Men (n=109)		P
Political-level, *n (%)*							
Political situation makes it difficult to maintain adequate glycemic control	117	(39.5)	84	(44.9)	33	(30.3)	0.013
Discrimination makes it difficult to maintain adequate glycemic control	183	(61.8)	126	(67.4)	57	(52.3)	0.010

DAPI Diabetes in the Arab population in Israel, SBGM self blood glucose monitoring, DM diabetes mellitus, PA physical activity

Supplemental Table 4. Multivariable logistic regression model of factors associated with ever having a dietician consultation among DAPI survey respondents (n=296)

Factor	β	SE	OR	95% CL		P
Age (per 10-y increment)	-0.008	0.015	0.93	0.69	1.24	0.604
Female vs male	0.113	0.139	1.25	0.73	2.16	0.417
Education, years (per 5-y increment)	0.070	0.031	1.42	1.05	1.92	0.023
DM duration (per 5-y increment)	0.039	0.019	1.22	1.01	1.47	0.041
Ever visited DM specialist: yes vs no	0.680	0.134	3.89	2.31	6.58	<0.001

DAPI Diabetes in the Arab population in Israel, DM diabetes mellitus

C-statistic=0.71

Supplemental Table 5. Multivariable logistic regression model of factors associated with meeting the leisure physical activity recommendation (≥2.5 hrs moderate-to-vigorous activity/wk) among DAPI survey respondents (n=296)

Factor	β	SE	OR	95% CL		P
Age (per 10-y increment)	-0.009	0.021	0.91	0.60	1.39	0.666
Female vs male	0.338	0.216	1.97	0.84	4.59	0.118
Years of education (per 5-y increment)	0.122	0.055	1.84	1.08	3.16	0.026
DM duration (per 5-y increment)	-0.098	0.039	0.61	0.42	0.90	0.012
No physical limitations to exercising	1.026	0.244	7.78	2.99	20.23	<0.001
Glycemic control not disrupted by *za'al*[*]	0.485	0.231	2.64	1.07	6.52	0.036

DAPI Diabetes in the Arab population in Israel, DM diabetes mellitus

[*]Sadness/anger/distress

C-statistic=0.84

Supplemental Table 6. Multivariable logistic regression model of factors associated with reporting economic barriers to some aspect of diabetes management among DAPI survey respondents (n=296)

Factor	β	SE	OR	95% CI		P
Age (per 10-y increment)	-0.027	0.018	0.97	0.94	1.01	0.119
Female vs male	0.355	0.160	2.03	1.09	3.80	0.026
Have disability: yes vs no	0.486	0.184	2.43	1.28	4.64	0.008
Currently unemployed vs employed	0.889	0.329	2.64	1.28	5.44	0.007

DAPI Diabetes in the Arab population in Israel

C-statistic=0.71

Supplemental Table 7. Multivariable logistic regression model of factors associated with moderate-to-severe depression symptoms (PHQ-9 score≥10) among DAPI survey respondents (n=296)

Factor	β	SE	OR	95% CL		P
Age (per 10-y increment)	-0.031	0.017	0.74	0.53	1.02	0.067
Female vs male	0.407	0.152	2.26	1.25	4.09	0.007
Have disability: yes vs no	0.498	0.144	2.71	1.54	4.77	0.001
Currently unemployed vs employed	0.701	0.232	4.07	1.64	10.10	0.003
DM duration (per 5-y increment)	0.061	0.021	1.36	1.11	1.66	0.003
HbA1c test result (per 1% increment)	0.184	0.080	1.20	1.03	1.41	0.022

PHQ-9 Patient Health Questionnaire-9, DAPI Diabetes in the Arab population in Israel, DM diabetes mellitus

C-statistic=0.75
